# Herbal formula YGJDSJ inhibits anchorage-independent growth and induces anoikis in hepatocellular carcinoma Bel-7402 cells

**DOI:** 10.1186/s12906-018-2083-2

**Published:** 2018-01-16

**Authors:** Bing Hu, Tong Zhang, Hong-Mei An, Jia-Lu Zheng, Xia Yan, Xiao-Wei Huang

**Affiliations:** 1 Department of Oncology, Longhua Hospital, Shanghai University of Traditional Chinese Medicine, Shanghai, 200032 People’s Republic of China; 2 Institute of Traditional Chinese Medicine in Oncology, Shanghai Academy of Traditional Chinese Medicine, Shanghai, 200032 People’s Republic of China; 3 Experiment Center for Teaching and Learning, Shanghai University of Traditional Chinese Medicine, Shanghai, 201204 People’s Republic of China; 4 Department of Science & Technology, Longhua Hospital, Shanghai University of Traditional Chinese Medicine, Shanghai, 200032 People’s Republic of China

**Keywords:** Hepatocellular carcinoma, Chinese herb, Anoikis, Caspases, Reactive oxygen species, Protein tyrosine kinase 2

## Abstract

**Background:**

Based on clinical medications and related studies, we established a Yang-Gan Jie-Du Sang-Jie (YGJDSJ) herbal formula for hepatocarcinoma treatment. In present study, we evaluated the anti-cancer potential of YGJDSJ on suspension-grown human hepatocellular carcinoma Bel-7402 cells.

**Methods:**

Bel-7402 cells were cultured in poly(2-hydroxyethyl methacrylate) (poly-HEMA) coated plates and treated with YGJDSJ. Anchorage-independent cell growth was detected by cell Counting Kit-8 (CCK-8) assay and soft agar colony formation assay. Anoikis was detected by ethdium homodimer-1 (EthD-1) staining and flow cytometry analysis. Caspases activities were detected by the cleavage of chromogenic substrate. Reactive oxygen species (ROS) was detected by 2′,7′-dichlorofluorescin diacetate (DCFH-DA) staining. Protein expression and phosphorylation was identified by western blot. Protein expression was knocked-down by siRNA.

**Results:**

YGJDSJ inhibited the proliferation of Bel-7402 cells in poly-HEMA coated plates and anchorage-independent growth of Bel-7402 cells in soft agar. YGJDSJ also induced anoikis in Bel-7402 cells as indicated by EthD-1 staining and flow cytometry analysis. YGJDSJ activated caspase-3, − 8, and − 9 in suspension-grown Bel-7402 cells. The pan-caspase inhibitor Z-VAD-FMK significantly abrogated the effects of YGJDSJ on anoikis in suspension-grown Bel-7402 cells. In addition, YGJDSJ increased ROS in suspension-grown Bel-7402 cells. The ROS scavenger N-acetyl-L-cysteine (NAC) partially attenuated YGJDSJ-induced activation of caspase-3, − 8 and − 9 and anoikis in suspension-grown Bel-7402 cells. Furthermore, YGJDSJ inhibited expression and phosphorylation of protein tyrosine kinase 2 (PTK2) in suspension-grown Bel-7402 cells. Over-expression of PTK2 significantly abrogated YGJDSJ induced anoikis.

**Conclusions:**

YGJDSJ inhibits anchorage-independent growth and induce caspase-mediated anoikis in Bel-7402 cells, and may relate to ROS generation and PTK2 downregulation.

## Background

Hepatocarcinoma is one of the most common malignancies worldwide, and ranks second and sixth as the cause of cancer deaths in men and women, respectively [1]. The treatment options for hepatocarcinoma mostly include surgery, transhepatic artery chemoembolization (TACE), and targeted therapy. Surgery, comprising either hepatectomy and/or liver transplantation, is the only treatment that can possibly cure hepatocarcinoma. However, only patients with early-stage hepatocarcinoma are eligible for curative surgery. The prognosis for patients with middle and advanced stages of hepatocarcinoma, especially metastatic hepatocarcinoma, remains poor [2, 3]. Hepatocarcinomas tend to metastasize to the lungs, bones, adrenal glands, and other distant organs via blood circulation. Hepatocarcinomas can also metastasize to the lymph nodes via circulating lymph, including portal lymph nodes, periaortic lymph nodes, and supraclavicular lymph nodes [4, 5]. Cancer metastasis is closely associated with anoikis resistance [6, 7].

Anoikis, also known as detachment-induced apoptosis, is the programmed cell death of epithelial cells (including cancer cells) following their detachment from the extracellular matrix (ECM) [8]. Anoikis was first discovered by Frisch and Francis in 1994 [9]. Cancer cells possess a certain degree of resistance to anoikis due to abnormal gene expression or activity that enables them to survive in circulating blood, lymph, or other liquid media, and this ultimately causes metastasis in distant tissues [6, 10]. However, cancer cells can undergo anoikis upon appropriate drug treatment. For example, fucoxanthinol, 5-azacytidine and foretinib can promote anoikis in cancer cells via different mechanisms [11–13]. Promoting cancer cell anoikis has become an important strategy for preventing cancer metastasis [14, 15].

Traditional Chinese medicine (TCM) is an important biomedical resource and plays important role in the treatment of hepatocarcinoma [16]. Based on clinical medications and TCM principles, we have established a herbal formula, Yang-Gan Jie-Du Sang-Jie (YGJDSJ), for hepatocarcinoma treatment. YGJDSJ comprises several Chinese herbs including the fruits of *Ligustrum lucidum* Ait. (Nü-zhen-zi), *Duchesnea indica* (Andr.) Focke (She-Mei), *Solanum nigrum* L. (Long-Kui), *Euphorbia helioscopia L.* (Ze-Qi), the root of *Ranunculus ternatus* Thunb. (Mao-Zhua-Cao), the root of *Curcuma wenyujin* Y. H. Chen et C. Ling (Yü-Jin) and the root of *Polygonum cuspidatum* Sieb. et Zucc. (Hu-Zhang). Most herbs in YGJDSJ have demonstrated anti-cancer effects in various cancer cells [16, 17]. In the present study, the effects and possible mechanism of YGJDSJ on anchorage-independent growth and anoikis of hepatocarcinoma cells were evaluated.

## Methods

### Chemicals and reagents

DMEM medium and fetal bovine serum was obtained from Hyclone (Logan, UT). Cell Counting Kit-8 (CCK8) was from Dojindo (Kumamoto, Japan). Caspases activities detection kits, 2′,7′-dichlorofluorescin diacetate (DCFH-DA), and N-acetyl-L-cysteine (NAC) were purchased from Beyotime (Haimen, China). Z-VAD-FMK was from R&D Systems (Minneapolis, MN). Antibodies against protein tyrosine kinase 2/focal adhesion kinase (PTK2/FAK), p-PTK2 and glyceraldehyde-3-phosphate dehydrogenase (GAPDH) were the product of Cell Signaling Technology (Danvers, MA). Poly(2-hydroxyethyl methacrylate) (poly-HEMA) was produced by Sigma-Aldrich (St. Louis, MO). CytoSelect™ 24-Well Anoikis Assay kit was provided by Cell Biolabs (San Diego, CA). Caspase-3, 8 and 9 activity assay kits were provided by Beyotime Institute of Biotechnology (Haimen, China).

### Cell culture

Human hepatocellular carcinoma Bel-7402 cells were obtained from Cell Bank of Type Culture Collection of Chinese Academy of Sciences. Bel-7402 cells were grown in DMEM medium with 10% FBS and 1% Pen-Strep, and maintained at a 37 °C in a humidified incubator with a 5% CO_2_ atmosphere. All the cell treatment was did in 10% FBS condition.

### Herb preparation

The main herbs in YGJDSJ formula (Chinese patent ZL201110145109.0) are the fruits of *L. lucidum* Ait. (Nü-zhen-zi) 12 g, *D. indica* (Andr.) Focke (She-Mei) 15 g, *S. nigrum* L. (Long-Kui) 15 g, *Scutellaria barbat*a D. Don (Ban-Zhi-Lian) 30 g, *E. helioscopia L.* (Ze-Qi) 15 g, the root of *R. ternatus* Thunb. (Mao-Zhua-Cao) 15 g, the root of *C. wenyujin* Y. H. Chen et C. Ling (Yü-Jin) 15 g and the root of *P. cuspidatum* Sieb. et Zucc. (Hu-Zhang) 15 g. The doses of these herbs were based on clinical medication.

All those herbs were from Longhua Hospital according to the original proportion. Herb extraction was performed as described previously [18, 19]. Briefly, herbs were extracted twice with an 8-fold volume of boiling distilled water for 1 h and the aqueous extracts were collected. The collected aqueous extracts were combined, filtered, centrifuged twice at 12,000 rpm for 30 min at 4 °C, and the supernatants were collected. The supernatants were then mixed with an equal volume of ethanol and kept at 4 °C overnight, centrifuged at 12,000 rpm for 30 min at 4 °C and the supernatants were collected and lyophilized. Subsequently, the ethanol extracts were dissolved in DMEM medium (400 mg/ml), sequentially passed through 0.45 μm and 0.22 μm filters for sterilization, and stored at − 20 °C until further use.

### Anchorage-independent growth assay

Poly-HEMA, a non-toxic polymer of 2-hydroxyethyl methacrylate, was used for anchorage-independent cell growth in vitro because of its ability to reduce the adhesivity of plastic cell culture plates. Bel-7402 cells in logarithmic growth phase were seeded into poly-HEMA coated 96-well plate (8 × 10^3^ cells/well). After 24 h cells were exposed to various doses of YGJDSJ or equal volume of DMEM for 24 h, and cell viability was evaluated by using the CCK-8 assay according to the manufacturer’s instructions. The cell survival rate was calculated as follows: cell survival rate (%) = (experimental OD value/control OD value) × 100%.

For the soft agar colony formation assays, 2 × 10^4^ log-phase Bel-7402 cells were seeded and grown on a plate containing 1% base agar and 0.6% top agar, and exposed to different concentrations of YGJDSJ or equal volume of DMEM twice a week for 2 weeks and incubated at 37 °C in a humidified incubator with a 5% CO_2_ atmosphere. Colonies were stained with crystal violet a counted under a dissecting microscope. The inhibition of colony formation was calculated as follows: inhibition (%) = [(control colonies - experimental colonies)/control colonies] × 100%.

### Anoikis assay

Anoikis was detected by CytoSelect™ 24-Well Anoikis Assay kit according to the manufacturer’s instructions. Briefly, log-phase Bel-7402 cells (4 × 10^4^ cells/well) were inoculated in poly-HEMA coated 24-well plate. On the second day, the cells were exposed to different dose of YGJDSJ or equal volume of DMEM for 24 h, and stained with ethidium homodimer (EthD-1) at 37 °C for 1 h. The presence of red EthD-1 fluorescence was monitored under a fluorescence microscope and measured with a fluorescence microplate reader (excitation wavelength 525 nm, emission wavelength 590 nm). EthD-1 is a high-affinity fluorescent nucleic acid dye, which can only penetrate membrane damaged dead cells and produces red fluorescence upon binding to nucleic acids, and thus be used to detect cell death in suspension (anoikis).

### Flow cytometric analysis

For apoptosis identification, 2 × 10^5^ log-phase Bel-7402 cells were seeded in poly-HEMA coated 6-well plate. On the second day, cells were treated with different concentrations of YGJDSJ or equal volume of DMEM for 24 h. YGJDSJ treated Bel-7402 cells were collected, stained with Annexin V-FITC and PI as recommended by the manufacturer, and detected in a FACScalibour flow cytometer (Becton Dickinson).

### Caspase activity assay

After treatment with different concentration of YGJDSJ, caspase-3, 8 and 9 activities were measured by the cleavage of the specific chromogenic substrate Ac-DEVD-pNA, Ac-IETD-pNA and Ac-LEHD-pNA respectively. The cleavaged yellow pNA are positive correlation with caspase-3, 8 and 9 activities. The presence of yellow pNA was detected with a microplate reader at a wavelength of 405 nm. The results are expressed as the fold change in comparison with the control group. For caspases inhibition, Bel-7402 cells pretreated with Z-VAD-FMK (50 μmol/L, 2 h) were incubated with YGJDSJ for another 24 h.

### Measurement of intracellular reactive oxygen species (ROS)

Intracellular ROS production was detected by DCFH-DA staining. DCFH-DA is cleaved intracellularly by nonspecific esterases to form DCFH, which is further oxidized by ROS to form the fluorescent compound DCF [20]. Log-phase Bel-7402 cells (4 × 10^4^ cells/well) were seeded in poly-HEMA coated 24-well plate. On the second day, the cells were exposed to different dose of YGJDSJ or equal volume of DMEM for 24 h, and stained with DCFH-DA at 37 °C for 20 min in the dark. The presence of DCF fluorescence was observed under a fluorescence microscope and quantitated with a fluorescence microplate reader at excitation wavelength of 488 nm and emission wavelength of 525 nm. For ROS inhibition, cells were pretreated with NAC (50 mmol/L for 2 h), followed by desired YGJDSJ treatment.

### Western blot

Western blots were performed as described previously [21, 22]. Briefly, collected cells were lysed and subjected to 8–12% SDS-PAGE, and transferred onto a nitrocellulose membrane (Amersham Biosciences, Buckinghamshire, UK). The transferred membrane were blocked with 5% non-fat milk, washed, and probed with the indicated antibodies. Blots were then washed and incubated with IRDye 700- or IRDye 800-conjugated secondary antibodies (Rockland Immunochemicals, Gilbertsville, PA, USA), and visualized in Odyssey Infrared Imaging System (LI-COR Biosciences, Lincoln, NE, USA).

### Plasmid transfection

For plasmid transfection, Bel-7402 cells were cultured on 6-well plate to 90–95% confluence, and 4.0 μg recombinant human PTK2 eukaryotic expression plasmid or control empty vector (Genechem, Shanghai, China) were introduced into the cells by Lipofectamine™ 2000 according to the manufacturer’s recommendations. After 24 h of transfection, cells were subjected to suspension-culture, YGJDSJ (200 μg/ml) treatment for 24 h, western blot and anoikis assay.

### Statistical analyses

Results are expressed as means ± standard deviation of at least two independent experiments, each conducted in triplicate. Differences between control and YGJDSJ treatment were analyzed by one-way ANOVA. Differences were considered significant at *P* ≤ 0.05.

## Results

### YGJDSJ inhibits anchorage-independent growth of Bel-7402 cells

**Fig. 1:**
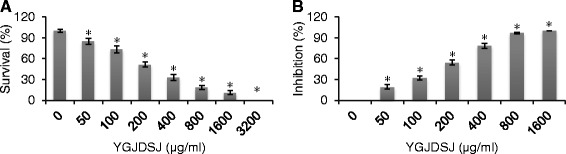
YGJDSJ inhibited anchorage-independent growth of Bel-7402 cells. **a** Bel-7402 cells were cultured in poly-HEMA coated 96-well plate and treated with different concentrations of YGJDSJ for 24 h, cell viability was evaluated by CCK-8 assay. **b** Bel-7402 cells were treated with different dose of YGJDSJ twice a week for 2 weeks in soft-agar colony formation assay. Data shown are representative of three independent experiments. *P < 0.01, versus control group

Poly-HEMA-coated culture plates were used to observe the effects of YGJDSJ on anchorage-independent growth in Bel-7402 cells. The results show that YGJDSJ inhibited proliferation of Bel-7402 cells in the poly-HEMA coated plates in a dose-dependent manner (*P* < 0.01) (Fig. 1a).

The effects of YGJDSJ on long-term anchorage-independent Bel-7402 cell growth were further observed using soft agar colony formation assays. The results show that YGJDSJ inhibits colony formation of Bel-7402 cells in a dose-dependent manner (*P* < 0.01) (Fig. 1b). These observations suggest that YGJDSJ can inhibit anchorage-independent growth in Bel-7402 cells.

### YGJDSJ induces anoikis in Bel-7402 cells

**Fig. 2:**
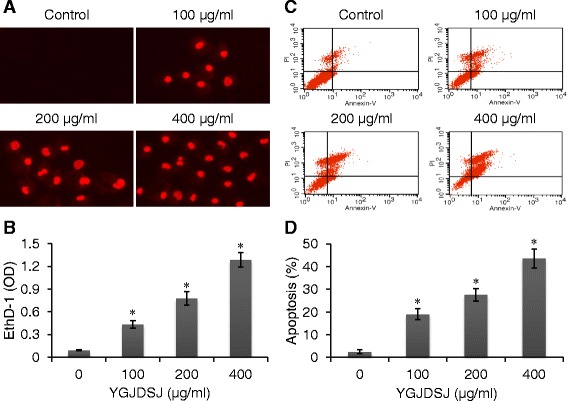
YGJDSJ induced anoikis in Bel-7402 cells. Suspension-grown Bel-7402 cells were treated with different dose of YGJDSJ for 24 h, stained with EthD-1, and observed under fluorescence microscope (× 200) (**a**), and quantitated with a fluorescence microplate reader (**b**). YGJDSJ treated or untreated Bel-7402 cells were stained with Annexin V-FITC/PI, analyzed in FACScalibour flow cytometer (**c**), and expressed as mean ± SD (**d**). Data illustrated are from three separate experiments. *P < 0.01, versus control group

Anoikis in Bel-7402 cells was detected with a commercial kit, in which the 24-well culture plate was coated with poly-HEMA, and EthD-1 fluorescence labeling was used for anoikis detection. The results show that after YGJDSJ treatment, Bel-7402 cells absorbed EthD-1 and emitted red fluorescence, which suggesting YGJDSJ induced anoikis in Bel-7402 cells (Fig. 2a and b). Further Annexin V-FITC/PI double labeling and flow cytometry analysis confirmed that YGJDSJ significantly induced apoptosis in suspension-grown Bel-7402 cell in a dose-dependent manner (*P <* 0.01) (Fig. 2c and d). These results suggest that YGJDSJ induced anoikis in Bel-7402 cells.

### YGJDSJ activates caspase activity in Bel-7402 cells

**Fig. 3:**
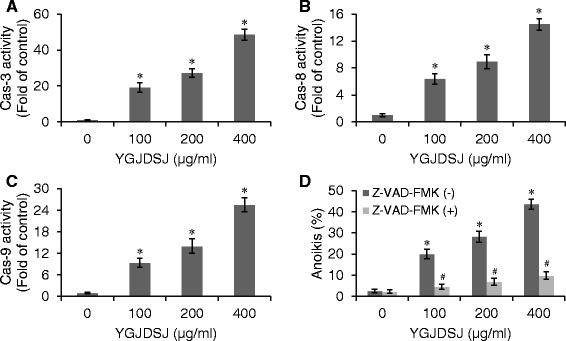
YGJDSJ activated caspases in Bel-7402 cells. After 24 h YGJDSJ (100–400 μg/ml) treatment, caspase-3 (**a**), caspase-3 (**b**) and caspase-9 (**c**) activities in suspension-cultured Bel-7402 cells were detected as described in Materials and Methods. Caspases activities were expressed as fold activation over control. **d** suspension-cultured Bel-7402 cells were pretreated with Z-VAD-FMK (50 μmol/L) for 2 h before treatment with YGJDSJ for 24 h, stained with Annexin V-FITC/PI and analyzed by flow cytometry. Data presented are from three separate experiments. *P < 0.01, versus control group; ^#^*P* < 0.01, versus corresponding dose of YGJDSJ treated Z-VAD-FMK (−) group

Similar to that in apoptosis, anoikis is also mediated by the caspase cascade [6, 10, 23]. In this study, the effects of YGJDSJ herbs on caspase activity were detected by commercial kits. As shown in Fig. 3, YGJDSJ significantly activated caspase-3, − 8, and − 9 in suspension-grown Bel-7402 cells in a dose-dependent manner (*P <* 0.01) (Fig. 3a-c). In addition, Z-VAD-FMK, a pan-caspase inhibitor, significantly abrogated the effect of YGJDSJ on anoikis (*P <* 0.01) (Fig. 3d), which suggests that the effect of YGJDSJ on anoikis in Bel-7402 cells is dependent on caspases.

### YGJDSJ induces ROS production in Bel-7402 cells

**Fig. 4:**
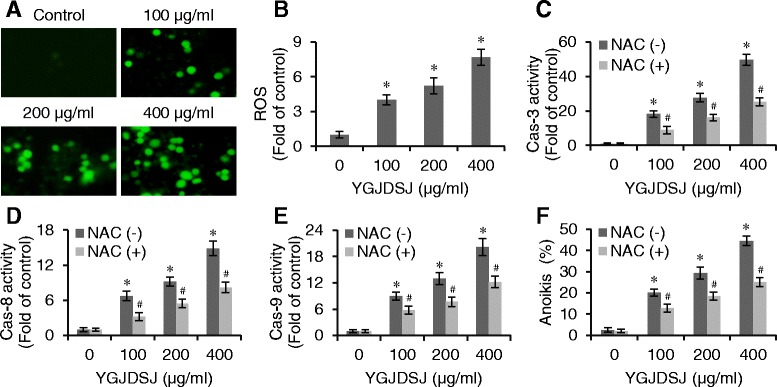
YGJDSJ increased ROS level in Bel-7402 cells. After 24 h YGJDSJ (100–400 μg/ml) treatment, intracellular ROS production in suspension-cultured Bel-7402 cells was stained with DCFH-DA, observed under fluorescence microscope (× 200) (**a**), quantitated with a fluorescence microplate reader and expressed as fold of control (**b**) Suspension-cultured Bel-7402 cells were pretreated with NAC (50 mmol/L for 2 h) for ROS inhibition, followed by YGJDSJ (100–400 μg/ml) treatment for 24 h, and subjected to caspase-3 (**c**), caspase-8 (**d**) and caspase-9 (**e**) activities and anoikis detection (**f**). Caspases activities were expressed as fold activation over control. Data shown are representative of three independent experiments. *P < 0.01, versus control group; ^#^*P* < 0.01, versus corresponding dose of YGJDSJ treated NAC (−) group.

It has been reported that ROS is an important mediator of anoikis and its levels increase with caspase activation [24]. In the present study, DCFH-DA staining was used to detect the ROS level. The results show that YGJDSJ significantly induced ROS generation in anchorage-independent grown Bel-7402 cells in a dose-dependent manner (*P <* 0.01) (Fig. 4a and b).

ROS generation was further blocked by NAC to address its role in caspases activation and anoikis. As shown in Fig. 4c-e, blocking ROS production with NAC antagonized the effect of YGJDSJ on caspase-3, − 8, and − 9 activation in Bel-7402 cells (*P <* 0.01). NAC also attenuated YGJDSJ induced anoikis in Bel-7402 cells (Fig. 4f) (*P <* 0.01). These observations suggest that ROS contributed to the effects of YGJDSJ on caspases activation and anoikis induction.

### YGJDSJ inhibits PTK2 expression

**Fig. 5:**
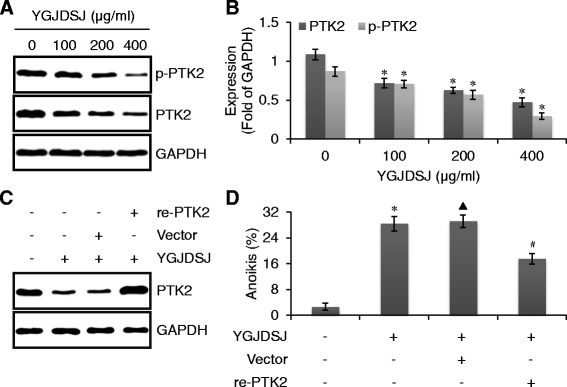
YGJDSJ inhibited PTK2 expression and phosphorylation in Bel-7402 cells. Suspension-cultured Bel-7402 cells were collected after YGJDSJ treatment, subjected to western blots using indicated antibodies (**a**), and the expression of proteins were expressed as fold of GAPDH (**b**). Bel-7402 cells were transfected with recombinant human PTK2 and empty vector, and subjected to suspension-culture, YGJDSJ (200 μg/ml) treatment for 24 h, western blots using indicated antibodies (**c**) and anoikis detection (**d**). *P < 0.01, versus control group; ^▲^*P* > 0.05, versus control YGJDSJ group; ^#^*P* < 0.01, versus YGJDSJ treated vector group

Tumor cells that detach from the ECM can escape from anoikis via PTK2 activation [25]. In this study, a western blot assay was used to detect the effects of YGJDSJ on the expression and phosphorylation of PTK2 in Bel-7402 cells. As shown in Fig. 5a and b, PTK2 was highly expressed and phosphorylated in anchorage-independent Bel-7402 cells and YGJDSJ inhibited both PTK2 expression and phosphorylation in a dose-dependent manner.

To examine whether down-regulation of PTK2 contribute to YGJDSJ induced anoikis, a recombinant eukaryotic expression plasmid encoding full length of human PTK2 (re-PTK2) was transfected to Bel-7402 cells. The results showed that PTK2 was over-expressed in re-PTK2 transfected Bel-7402 cells (Fig. 5c). PTK2 over-expression partially but significantly abrogated YGJDSJ induced anoikis (*P* < 0.01) (Fig. 5d). These observations suggested PTK2 down-regulation contributed to YGJDSJ-induced anoikis.

## Discussion

YGJDSJ formula was established in accordance with TCM theories and clinical medications [16, 17, 26]. We observed the basic TCM pathogenesis of liver cancer is liver Yin-deficiency and cancerous toxicity, and proposed a new therapeutic principle for liver cancer treatment, which is nourishing the liver-Yin (Yang-Gan, YG), detoxifying (Jie-Du, JD) and resolving tumor masses (Sang-Jie, SJ). Chinese herbs commonly medicated in our clinical practice with YG, JD and SJ efficacy were selected and combined as an YGJDSJ formula.

Most herbs in YGJDSJ have been confirmed to have anti-cancer effects. *L. lucidum* Ait. fruit (Nü-zhen-zi) is one of the most frequently used herbs in liver cancer treatment. *L. lucidum* Ait. fruit (Nü-zhen-zi) can inhibit proliferation, activate caspase-3, − 8 and − 9 to induce apoptosis and inhibit RB phosphorylation to promote cell senescence in hepatocellular carcinoma cells [19]. *L. lucidum* Ait. fruit (Nü-zhen-zi) in YGJDSJ was use for nourishing the liver-Yin.

*S. nigrum* L. (Long-Kui) can induce apoptosis and autophagy, and arrest the cell cycle in G2/M phase in hepatocellular carcinoma cells [27, 28]. In ovarian cancer, *D. indica* (Andr.) Focke (She-Mei) can promote cell apoptosis, arrest the cell cycle in the S phase, and inhibit tumor growth [29]. *S. barbata* D. Don (Ban-Zhi-Lian) can induce apoptosis via the mitochondrial pathway in liver cancer cells [30]. *S. nigrum* L. (Long-Kui), *D. indica* (Andr.) Focke (She-Mei) and *S. barbata* D. Don (Ban-Zhi-Lian) in YGJDSJ were used for detoxifying.

*E. helioscopia L.* (Ze-Qi) can promote apoptosis as well as inhibit growth, invasion, and metastasis of hepatocellular carcinoma [31, 32]. *C. wenyujin* Y. H. Chen et C. Ling (Yü-Jin) extracts can inhibit proliferation of colorectal carcinoma cells [33]. *P. cuspidatum* Sieb. et Zucc. (Hu-Zhang) shows anti-cancer effects against oral carcinoma, hepatocellular carcinoma, melanoma, and other tumor cells [18, 34, 35]. *E. helioscopia L.* (Ze-Qi), *R. ternatus* Thunb. (Mao-Zhua-Cao), *C. wenyujin* Y.H.Chen et C.Ling (Yü-Jin) and *P. cuspidatum* Sieb. et Zucc. (Hu-Zhang) in YGJDSJ were applied for resolving tumor masses. Thus, YGJDSJ is a modern herbal formula with anti-cancer effect in line with TCM theory. Herbs in YGJDSJ coordinate with each other in the aspect of traditional TCM efficacy. However, the additive or synergistic pharmacological effects of these herbs need further investigation.

*P. cuspidatum* Sieb. et Zucc. (Hu-Zhang) and *D. indica* (Andr.) Focke (She-Mei) have demonstrated anoikis-inducing effects in cancer cells [18, 36]. Anokis is usually investigated in cell models. Poly-HEMA coating and soft agar colony formation assays are classical anchorage-independent growth models, in which cell grow in a suspension and/or anchorage-independent manner [9, 18, 37, 38]. In the present study, it was shown that YGJDSJ inhibited the growth of Bel-7402 cells in poly-HEMA-coated plates and soft agar, which suggests that YGJDSJ can inhibit the anchorage-independent growth of Bel-7402 cells. Further EthD-1 staining and flow cytometric analysis showed that YGJDSJ could induce anoikis in Bel-7402 cells.

Anoikis is a special type of apoptosis, which is also mediated by the caspase cascade [6, 10, 23, 39]. Cells detached from the ECM can activate caspase-9 or − 8 via intrinsic or extrinsic pathways, thereby activating the apoptosis-executing protease caspase-3 and inducing anoikis. Results of this study revealed that YGJDSJ activated caspase-3, − 8, and − 9 in suspension-grown Bel-7402 cells. Z-VAD-FMK, a pan-caspase inhibitor, could abrogate the effect of YGJDSJ on anoikis in Bel-7402 cells. These findings suggest that YGJDSJ can induce Bel-7402 cell anoikis in a caspases-dependent manner via intrinsic and extrinsic apoptotic pathways.

ROS, including oxygen ions, peroxides, and oxygen-containing free radicals, is produced by cells during aerobic metabolism. High level of ROS can trigger apoptosis via intrinsic and/or extrinsic pathways [40, 41]. Natural products, such as *P. cuspidatum* Sieb. et Zucc. (Hu-Zhang), emodin, curcumin, and grape seed extracts, can induce anoikis in cancer cells by increasing intracellular ROS levels [11, 18, 42, 43]. In the present study, it was found that YGJDSJ induced ROS generation in Bel-7402 cells. In addition, ROS scavenger NAC could antagonize the effects of YGJDSJ on caspase-3, − 8, and- 9, and on anoikis in Bel-7402 cells. These findings suggest that ROS generation contributed to YGJDSJ activated caspases and induced anoikis.

Epithelial cells, including epithelial cancer cells, adhere to the ECM, form focal adhesion plaques mediated by integrin, and thereby promote PTK2 conformational changes and activation; this results in the production of survival signals for cell growth and proliferation through downstream signal transduction [23, 44, 45]. Detachment from ECM will result in anoikis. Cancer cells can escape anoikis by modulating PTK2 activity [25]. On the other hand, down-regulation of PTK2 expression can promote cancer cell anoikis [46, 47]. Results from this study demonstrate that YGJDSJ herbs could inhibit expression and phosphorylation of PTK2, and PTK2 overexpression could attenuate the effect of YGJDSJ on anoikis in Bel-7402 cells, suggesting that down-regulation of PTK2 contributes to YGJDSJ-induced anoikis.

It has been reported H_2_O_2_ or AKT inhibitor SC66 induced ROS can inhibit FAK (PTK2) phosphorylation [48, 49]. On the other hand, FAK inhibitor MSN-HCD may upregulate ROS level in glioma cells [50]. In addition, depression of FAK by shRNA induces apoptosis in rat osteosarcoma cells through activation of caspase-3, 7 and 9 [51]. Loss of Rb-E2F by displacement of promoters induces apoptosis through inactivation of FAK and activation of caspase-8 [52]. Doxazosin induces anoikis in prostate cancer cells via activation of caspase-3 and a reduction of FAK [53]. These observations suggest that YGJDSJ induced ROS and inhibited FAK may contribute to each other and both contribute to caspases activation.

## Conclusion

In summary, the present study showed that YGJDSJ inhibited suspension growth of Bel-7402 cells. YGJDSJ increased ROS generation, activated caspase-3, − 8, and − 9, and down-regulated PTK2 and thus induced anoikis in Bel-7402 cells. Since anoikis is related to cancer metastasis and cell survival in blood or lymphatic circulation, the effects of YGJDSJ against hepatocarcinoma metastasis and hepatocarcinoma cells in blood or lymphatic circulation, such as circulating tumor cells, are worthy of further study.

## Abbreviations

CCK-8: Cell counting kit-8
DCFH-DA: 2′,7′-dichlorofluorescin diacetate
DMEM: Dulbecco’s modified Eagle’s medium
ECM: Extracellular matrix
EthD-1: Ethidium homodimer-1
FAK: Focal adhesion kinase
FBS: Fetal bovine serum
GAPDH: Glyceraldehyde-3-phosphate dehydrogenase
NAC: N-acetyl-L-cysteine
poly-HEMA: poly(2-hydroxyethyl methacrylate)
PTK2: Protein tyrosine kinase 2
ROS: Reactive oxygen species
TACE: Transhepatic artery chemoembolization
TCM: Traditional Chinese medicine
YGJDSJ: Yang-Gan Jie-Du San-Jie herbal formula
